# The use of prednisolone versus dual-release hydrocortisone in the treatment of hypoadrenalism

**DOI:** 10.1530/EC-20-0473

**Published:** 2021-01-06

**Authors:** Sirazum Choudhury, Tricia Tan, Katharine Lazarus, Karim Meeran

**Affiliations:** 1 Endocrinology and Investigative Medicine, Department of Metabolism, Digestion and Reproduction, Imperial College London, Commonwealth Building, London, UK; 2 Department of Endocrinology, Imperial College Healthcare NHS Trust, London, UK

**Keywords:** Adrenal, Hypoadrenalism, Adrenal insufficiency, Glucocorticoid replacement, Pituitary

## Abstract

The introduction of adrenocortical extract in 1930 improved the life expectancy of hyhpoadrenal patients, with further increases seen after the introduction of cortisone acetate from 1948. Most patients are now treated with synthetic hydrocortisone, and incremental advances have been made with optimisation of daily dosing and the introduction of multidose regimens. There remains a significant mortality gap between individuals with treated hypoadrenalism and the general population. It is unclear whether this gap is a result of glucocorticoid over-replacement, under-replacement or loss of the circadian and ultradian rhythm of cortisol secretion, with the risk of detrimental excess glucocorticoid exposure at later times in the day. The way forwards will involve replacement of the diurnal cortisol rhythm with better glucocorticoid replacement regimens. The steroid profile produced by both prednisolone and dual-release hydrocortisone (Plenadren), provide a smoother glucocorticoid profile of cortisol than standard oral multidose regimens of hydrocortisone and cortisone acetate. The individualisation of prednisolone doses and lower bioavailability of Plenadren offer reductions in total steroid exposure. Although there is emerging evidence of both treatments offering better cardiometabolic outcomes than standard glucocorticoid replacement regimens, there is a paucity of evidence involving very low dose prednisolone (2–4 mg daily) compared to the larger doses (~7.5 mg) historically used. Data from upcoming clinical studies on prednisolone will therefore be of key importance in informing future practice.

## Introduction

Between 1928 and 1938, patients with Addison’s disease had a 100% 5-year mortality (1). With the availability of glucocorticoid replacement therapy, initially with animal adrenocortical extract and later synthetic 11-deoxycorticosterone (2), and cortisone acetate from 1948, the prognosis of Addison’s disease improved vastly. Patients were no longer dying from adrenal failure, and generous doses of glucocorticoids were given to guard against adrenal crises. Whilst the era of synthetic glucocorticoids has ushered in longer life expectancies, the use of liberal doses has come at the cost of increased long-term cardiometabolic death (3).

Half a century later, a retrospective observational study in Sweden demonstrated an increased relative risk of mortality in patients with Addison’s disease compared to the general population, between 1987 and 2001 (4). The leading cause of death was cardiovascular disease, and specifically ischaemic heart disease. This was followed by malignancy, endocrine causes, respiratory causes and infectious diseases.

These findings were supported in a further study investigating the Swedish Addison’s disease population (5). There was an overall increased standardised mortality ratio (SMR) of 2.7 for all Addison’s patients compared to the general population. Again, cardiovascular disease was the commonest cause of death with malignancy coming second. Within malignancy, gastrointestinal tract cancers were the most prevalent followed by male genital cancers and non-melanoma skin cancers.

A Norwegian study demonstrated that whilst the SMR of all patients with Addison’s disease was not significantly elevated compared to the general population at 1.15, there was concern for patients diagnosed under the age of 40, who had a significantly higher SMR of 1.5 (6). Cardiovascular disease, adrenal failure and cancer emerged as the top three causes. Overall, males and females diagnosed with Addison’s disease could expect a life expectancy that is 3.2 and 11.2 years shorter, respectively, than their counterparts in the general population. The study did not, however, report the range of glucocorticoid doses used.

The EU-AIR registry includes secondary adrenal insufficiency and data from the UK, the Netherlands and Germany in addition to Sweden (7). In this more heterogeneous group, the main causes of death were cardiovascular disease (35%) and infection (15%). In an exclusively hypopituitary population in the USA, Mills and colleagues described a seven-fold increase in mortality when associated with adrenal insufficiency (8). Taken together, these data suggest that the mortality gap in hypoadrenalism is not just limited to primary disease.

## Possible causes of the mortality gap

The cause of the aforementioned mortality gap has not been fully elucidated. Studies suggest the causes may include excess exposure to glucocorticoid replacement, under-replacement and risk of acute adrenal failure, failure to replicate the diurnal and ultradian rhythm of cortisol leading to steroid exposure at detrimental times in the day and finally, differences in the biological actions of oral synthetic glucocorticoids versus endogenous cortisol.

Interrogation of the EU-AIR registry demonstrated a higher mortality of 1.5% in patients with secondary hypoadrenalism vs 1.0% in patients with primary disease over approximately 5 years (7). In the secondary disease cohort, it was clear that those who died were in fact receiving higher doses of glucocorticoid replacement treatment, 24.0 mg of hydrocortisone vs 19.3 mg in the secondary cohort that remained alive.

These findings suggest that even very small excesses of glucocorticoid replacement may contribute towards poorer mortality outcomes. Sherlock and colleagues interrogated a regionally held UK database containing information on patients with acromegaly, which included 178 patients receiving hydrocortisone for hypoadrenalism (9). Patients with acromegaly had an increased SMR of 1.7 compared to the general population, but those on hydrocortisone showed a significant positive correlation for increasing SMR with an increasing daily hydrocortisone dose. Patients receiving greater than 30 mg of hydrocortisone daily and between 25 and 30 mg daily, had a relative risk of mortality of 2.9 and 1.6, respectively, compared to patients with acromegaly in the absence of hypoadrenalism. Even regimens greater than 20 mg of hydrocortisone may be detrimental. Evidence from Swedish populations showed that secondary hypoadrenal patients receiving such doses had a 1.88-fold increase in mortality over approximately 13 years, compared to hypopituitary patients not requiring glucocorticoid replacement. Crucially, this was not the case for those taking daily doses of 20 mg or less (10).

Escalating doses of glucocorticoid replacement are associated with worsening cardiovascular risk factors. In a three-arm crossover study, ten patients with secondary hypoadrenalism took 15 , 20 and 30 mg of hydrocortisone for 6 weeks (11). Ambulatory arterial stiffness index scores were significantly lower when participants received 15 mg of daily hydrocortisone compared to 20 and 30 mg. When secondary hypoadrenal patients were treated with 0.4–0.6 mg/kg of hydrocortisone daily, systolic and diastolic blood pressures were higher than those seen when patients received 0.2–0.3 mg/kg over a 10-week period (12). In Addison’s disease, a comparison between patients on a median of 30 mg of hydrocortisone and healthy matched controls revealed increased hepatic adiposity on CT imaging, as well as a higher fasting triglycerides and lower HDL (13). These surrogate endpoints suggest that higher doses of glucocorticoid replacement induce a metabolic syndrome which likely drives an excess risk of cardiovascular disease.

Additional evidence suggests, however, that the mortality gap is not directly linked to excess glucocorticoid replacement. In a cross-sectional comparison between individuals with Addison’s disease in Sweden and South Africa, it was noted that patients in Sweden received higher doses of hydrocortisone, 33.0 mg per day compared to 24.3 mg per day in South Africa (14). Despite being well matched and the apparent lower glucocorticoid exposure, the South African cohort had a significantly higher total cholesterol, triglycerides, and LDL, indicating a worse cardiovascular risk phenotype. Although it is possible that the observations may be due to the two sample cohorts being taken from two distinctly homogenoeus populations with their own potential genetic and environmental differences, it is important to note that the timing of doses was not considered in the study.

## Loss of diurnal rhythm in autonomous cortisol secretion also increases mortality

Both autonomous cortisol secretion and oral glucocorticoid replacement therapy result in a mild excess of glucocorticoids and an altered diurnal cortisol rhythm, with supraphysiological levels particularly in the latter half of the day.

Autonomous adrenal cortisol secretion is a pathological state analogous to the proposed cause of the described mortality gap. The autonomous secretion is difficult to diagnose and detect as the excess cortisol is only slightly and not overtly raised (15). Cortisol profiles are flat, with obliteration of the physiological diurnal rhythm and morning cortisol levels may be normal. From 206 individuals followed up over 4.2 years, one study has demonstrated a 4- and 10-year reduction in life expectancy for men and women, respectively, in the UK (16). Cardiovascular disease and infectious causes were the top two causes of death. In an Italian population, patients with adrenal masses suspicious of autonomous secretion were compared to individuals with non-secreting adrenal incidentalomas (17). Those with suspicion of autonomous secretion, defined as incomplete suppression of cortisol to levels of 50–138 nmol/L after 1 mg dexamethasone suppression testing, had lower survival rates. A similarly designed Swedish study, reported greater mortality in patients with autonomous cortisol secretion (18).

### A question of timing?

The normal cortisol profile has been well established and is conserved between individuals (19). Cortisol levels in humans peak at awakening, with a second peak at lunch time, and a gradual decline in levels to an overnight nadir that rises again 2–4 h before waking. Disassociation of the cortisol concentration from the expected pattern for the time of day is detrimental (20). In ten healthy individuals who were subjected to a 28-h day for 7 days, there was a 6 and 22% rise in 3-h postprandial glucose and insulin levels, compared to baseline, respectively. This observation occurred independently of fasting glucose levels, when a 12-h misalignment between the participants’ circadian cycle and their behavioural cycle (or meal times) was achieved (21). Three individuals demonstrated impaired glucose tolerance in relation to meals, despite being normoglycaemic prior to the study suggesting acute insulin resistance. Mean arterial pressure was also elevated.

The disconnect between serum cortisol levels and the circadian clock maintained by all cells may be central to the adverse outcomes of shift work. It is well characterised that during shift work, there is a reversal of the diurnal cortisol rhythm, such that peak levels are seen at night, whilst individuals are awake (21, 22, 23). Charmandari *et al*. investigated peripheral blood mononuclear cells (PBMCs), sampling cells at 08:00 h and 20:00 h (24). PBMCs are easily obtainable cells that are representative of peripheral tissue. They showed a 2.8-fold greater acetylation of the glucocorticoid receptor (GR) in the morning than in the evening. With acetylation of the GR attenuating the transcriptional response of the cells to glucocorticoids, sensitivity to glucocorticoids is in inverse phase to the circadian cortisol profile. The lowest sensitivity was seen in the morning when cortisol peaks, and the highest in the evening, when cortisol wanes (25). Marked differences have already been observed in healthy individuals between glucocorticoid exposure in the morning vs the afternoon (26). Administration of 50 mg oral hydrocortisone was compared at 05:00 h and 17:00 h. The cortisol drug profiles and glucose handling parameters within the first 4 h were identical at both clock times. Between 04:00 h and 16:00 h, the peak glucose excursion, insulin secretory rates and serum insulin levels were significantly higher with the 17:00 h hydrocortisone dose as compared to the 05:00 h dose, indicating greater sensitivity to glucocorticoids later in the day.

**Figure 1:**
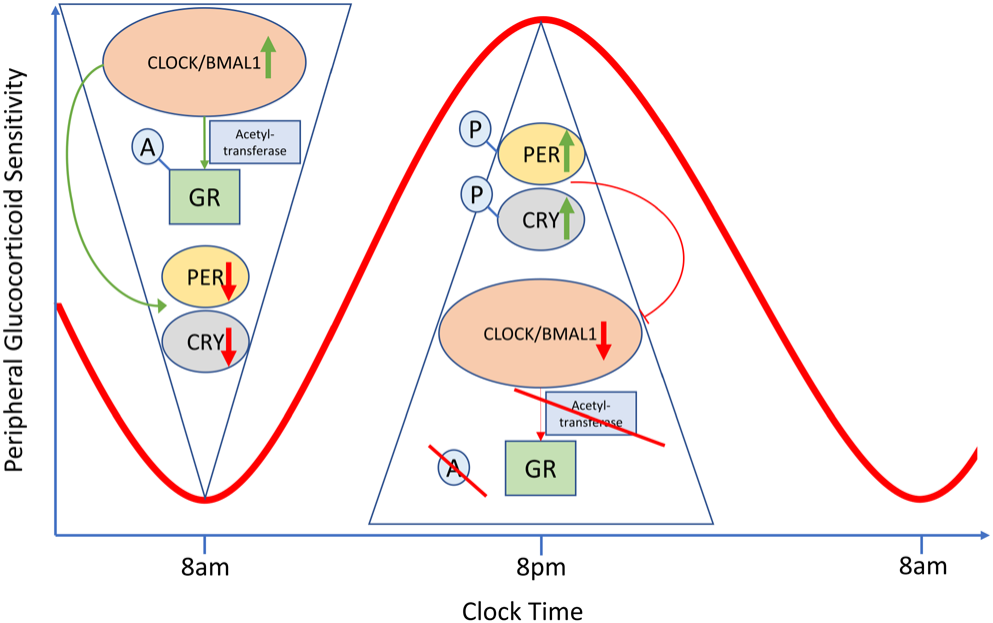
Model of glucocorticoid sensitivity in peripheral tissue as it fluctuates during the day (24, 25). Nadir sensitivity (peak resistance) is seen at 08:00 h, when cortisol secretion peaks. CLOCK/BMAL1 expression directly acetylates (A) the glucocorticoid receptor (GR), attenuating its function. CLOCK/BMAL1 also enhances the expression of the PER and CRY genes, although the mRNA expression is low at this time. At 20:00 h, the PER and CRY expression is high, and their phosphorylation (P) inhibits expression of CLOCK/BMAL1. The low CLOCK/BMAL1 expression, prevents acetylation of the GR, which in turn increases glucocorticoid sensitivity.

Acetylation of the GR can be influenced by clock genes. Clock genes represent the time keeping mechanism that exists in all human cells. The intracellular equipment responsible for cellular timekeeping involves a number of feedback and transcriptional loops. Central to this is circadian locomotor output cycle kaput (CLOCK) which dimerises with brain–muscle–arnt-like protein 1(BMAL1). The CLOCK/BMAL1 dimer in turn binds to enhancer sequences in the DNA of cells to promote transcription of period genes (PER1, PER2, PER3) and cryptochromes (CRY1 and CRY2) (25). The ‘master’ clock in the body is the suprachiasmatic nucleus (SCN) in the hypothalamus, which benefits from innervation from the retina, allowing entrainment by day–night cycle (27). It is signalling from the ‘master’ clock which informs the ‘slave’ clocks residing in all other tissues that maintain synchrony (25). In the hypothalamic-pituitary-adrenal (HPA) axis, the influence of the master clock is exerted by canonical endocrine signalling via arginine vasopressin (AVP) modification of pulsatile ACTH secretion, but there also exist non-endocrine pathways with neural signalling via the splanchnic innervation of the adrenals and a local circadian clock within the adrenal glands. Apart from measurable rhythms in cortisol, GR activity is influenced by its own rhythm. CLOCK influences acetyl-transferase activity, which is directly capable of acetylating and attenuating GR function (Fig. 1) (28). This is in keeping with results from Charmandari and colleagues, where CLOCK/BMAL1 expression was relatively higher at 08:00 h, at the same time that the GR was maximally acetylated and glucocorticoid sensitivity at its lowest (24).

In addition, glucocorticoids can also affect and manipulate the timekeeping machinery of peripheral cells. Cuesta *et al*. recruited 16 healthy males, collecting PBMCs at baseline and at 6 days after the participants had taken hydrocortisone 20 mg orally every evening, 10 h post awakening (29). They found that a single dose of hydrocortisone can provoke a significant increase in PER1 mRNA expression. Further, after 6 days, PER2 levels were found to be reduced in those who responded and to have phase shifted forwards, meaning the peak levels were 9 h later than baseline. PER3 demonstrated a single pre-awakening peak at baseline, but after 6 days, a new second peak in the evening was present. Taken together, these results indicate that a single dose of hydrocortisone 20 mg in the evening can alter the expression of clock genes in peripheral cells and may in turn modify the glucocorticoid sensitivity of these cells as a result.

## Bridging the mortality gap

The evidence presented suggests that the excess mortality seen in treated patients with adrenal insufficiency may be driven by glucocorticoid over-replacement, especially at times of increased sensitivity, such as the evening. The failure to mimic the circadian cortisol profile is central to these mechanisms. Standard-release hydrocortisone is the most common treatment used in the UK and Europe (30, 31). Its short half-life of 1.8 h mandates multiple doses per day (32, 33). The final dose exposes patients to the risk of having excess glucocorticoid in their blood at times in the day when it is potentially detrimental. As a result of its pharmacokinetic profile, oral hydrocortisone is inherently unable to mirror the circadian cortisol rhythm. The multiple dosing regimen can also result in incomplete dosing as patients may not always take hydrocortisone on time.

Continuous subcutaneous hydrocortisone infusion (CSHI) pumps, may offer a more physiological alternative cortisol replacement therapy (34), particularly for those unable to tolerate or absorb oral replacement. In an unblinded open-label feasibility study, an improvement in the vitality and physical functioning domains of the short form health survey (SF-36) measuring health related quality of life, was noted when patients were converted from oral hydrocortisone to CSHI (34). However, in a double-blind, placebo-controlled, randomised crossover trial comparing oral hydrocortisone and CSHI, there was no additional benefit seen with CSHI in subjective health scores (35). The use of CSHI requires patient training and engagement, necessitating education on pump use and maintenance. There is also a risk of local site infections and dislodgement with interruption of steroid delivery (36). The subjective health benefits of CSHI have not been conserved between studies and cardiovascular risk as assessed by anthropometric and biochemical markers, has not been adequately explored. As such, there are currently insufficient data from CSHI studies to conclude that the more physiological replacement offered translates into better long-term outcomes.

**Figure 2:**
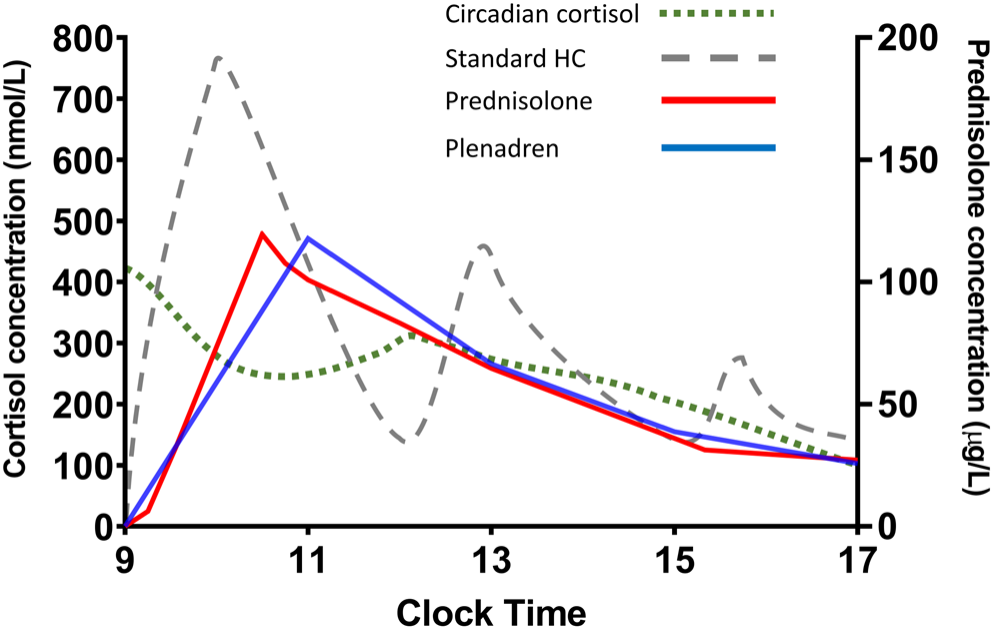
Serum glucocorticoid profiles of: 1-endogenous circadian cortisol (green dashed); 2-standard thrice daily hydrocortisone regimen (HC) (grey dotted); 3-prednisolone 4 mg once daily (red solid); 4-Plenadren 20 mg once daily (blue solid). (1) and (2) are plotted from data extracted from Mah *et al.* 2004 (64). (3) and (4) are generated from data collected at Imperial College Healthcare NHS Trust. Thrice daily hydrocortisone generates a peak and trough profile that both over- and under-shoot the normal cortisol profile. Plenadren and prednisolone generate a similar curve that is closer in morphology to the diurnal cortisol rhythm.

Dual-release hydrocortisone (herein referred to as Plenadren), and prednisolone both offer a once-daily solution to glucocorticoid replacement therapy. Apart from the convenience and improved adherence to treatment with once-daily dosing, both drugs produce a smoother plasma profile (Fig. 2) (37, 38). As a result, Plenadren and prednisolone may offer better alternatives to standard-release multidose hydrocortisone, which in turn may improve the mortality outcomes.

### Plenadren

Plenadren is a dual-release formulation of hydrocortisone containing both immediate release and sustained release hydrocortisone in a single tablet. Therefore it is designed to give a smoother glucocorticoid profile than standard- release hydrocortisone (39). It is available as 5 and 20 mg tablets. Data from a salivary cortisol study has shown that although morning peaks in hypoadrenal patients are equivalent to the overshoot associated with thrice daily cortisone acetate or standard hydrocortisone, Plenadren is able to generate afternoon cortisol levels that tend towards the levels seen in healthy controls (40).

In an open-label study, 64 primary hypoadrenalism patients took 3 months of thrice-daily hydrocortisone and once-daily Plenadren in a randomised crossover protocol. Patients were converted from their daily cumulative dose of thrice-daily hydrocortisone to the same total daily dose of Plenadren, administered once a day in the morning (38). Pharmacokinetic studies were performed in 18 patients. The area-under-the-curve (AUC) cortisol profile demonstrated a reduction of 19.4% in AUC_0–24 h_, for Plendaren compared to standard release hydrocortisone. There was a 6.4% increase in AUC_0–4 h_, with subsequent reductions of 30.5 and 58.8% for AUC_4–10 h_ and AUC_10–24 h,_ respectively. This amounts to both, a drop in total steroid exposure and a specific reduction in the amount of steroid exposure in the afternoon and evenings. There were significant decreases with Plenadren in body weight, systolic blood pressure and diastolic blood pressure although this was tempered slightly by a significant increase in heart rate and fasting triglycerides. Further possible benefits were seen, with reductions in HbA1c by 0.1% and increase of 6.1 µg/L in procollagen type 1 N-terminal propeptide (P1NP), a bone turnover marker of osteoblastic activity. Subjective quality of life outcomes also favoured Plenadren over standard hydrocortisone. Overall, Plenadren demonstrated superiority over thrice-daily hydrocortisone in term of cardiovascular risk factors and bone health markers.

In an extension of the aforementioned study, 55 of the original 64 patients continued on Plenadren for up to 24 months after the end of the randomised crossover phase. An additional 16 patients were recruited into this study, who had not participated in the crossover trial and took Plenadren for up to 18 months (41). The safety data from this study extension showed that patients on Plenadren experienced 18.6 serious adverse events (SAEs) per 100 patient-years, compared to 13.3 SAEs per 100 patient-years on standard hydrocortisone. Notably, gastroenteritis caused hospitalisation of 11 out of the 19 patients who experienced SAEs, possibly because the dual-release formulation is more vulnerable to malabsorption in the case of intercurrent gastroenteritis. After 18 months, there continued to be a significant reduction in weight by 1.4 kg, but no change in blood pressure or HbA1c, in contrast to the 3-month data. Despite the suggestion of an increased SAE rate from the extension study, further follow-up to 5 years has confirmed that there are no significant safety concerns with the use of Plenadren and that it remains generally well tolerated (42).

In a further study, 19 individuals with Addison’s disease were switched from 20 mg standard hydrocortisone in divided doses to Plenadren 20 mg once daily and evaluated over a 12-month period (43). Over 1 year, patients underwent a quarterly assessment, during which BMI, body weight and waist circumference followed an encouraging downward trend, with only waist circumference achieving significance. HbA1c was 2.0 mmol/mol lower at 1 year with Plenadren compared to baseline, with diabetic patients also showing improvement and lower insulin requirements. Although AddiQOL scores indicated better quality of life, the fatigue score was noted to have worsened with Plenadren. These findings are partly corroborated by a retrospective study of 49 patients with a mixture of primary and secondary hypoadrenalism, who were switched to Plenadren for a longer period of 36 months (44). Twenty-five participants were non-diabetic and 24 were prediabetic, amongst whom 30 were initially on hydrocortisone replacement and 19 on cortisone acetate. Overall, a significant reduction in BMI, waist circumference and HbA1c were observed in all patients. In addition to this, the participants with prediabetes also showed reductions in fasting insulin, insulin secretion over 2 h in response to an oral glucose tolerance test (OGTT) and an increase in both insulin sensitivity and HDL. No improvements were seen in blood pressure, and notably, 13 patients under the guidance of clinicians received a greater dose of Plenadren than expected.

There is also evidence of other metabolic benefits including improvement of hepatic steatosis (45). In 45 patients with secondary adrenal insufficiency, 25 of whom were already being treated with hydrocortisone and 20 yet to start replacement, Plenadren was administered for a 12-month period. At baseline, 31 individuals were diagnosed with steatosis on ultrasound imaging. At 12 months there were significant reductions in BMI, waist circumference, fasting insulin, insulin resistance according to homeostatic model assessment (HOMA-IR) with a corresponding increase in the insulin sensitivity. The hepatic steatosis index was noted to have significantly reduced in the cohort and the number of individuals with an index of greater than 36, came down from 33 to 11. No differences were detected in HbA1c or blood pressure, and six participants required a dose increase during the 12-month period.

The effects of Plenadren on blood pressure are difficult to interpret. Ten patients out of 17, stably replaced with cortisone acetate and diagnosed with adrenal insufficiency, were converted to Plenadren in a retrospective, case-control analysis (46). When patients were treated with Plenadren for 6 months, nocturnal diastolic pressure rose by 9 mmHg. As the relative potency of cortisone acetate and hydrocortisone is not clear, the relevance of this study to patients converting from standard release hydrocortisone to Plenadren may be limited.

A study retrospectively collected data on 14 patients who had dual energy X-ray absorptiometry (DEXA) imaging before and after switching to Plenadren (47). All patients were diagnosed with secondary hypoadrenalism, had been stable on cortisone acetate or hydrocortisone therapy for 12 months prior to the change, and had been on Plenadren for at least 2 years before the second DEXA scan. There was a significant increase in the bone mineral density in the lumbar spine and femoral neck, but not the total hip, independent of vitamin D status.

In the DREAM study, 46 patients with primary or secondary hypoadrenalism were switched from multiple daily doses of either cortisone acetate or hydrocortisone to Plenadren and were compared to 43 patients who continued on their standard regimen, as well as 25 healthy controls (48). Patients were tracked on their treatments over 24 weeks. Between the patient groups, the corrected change in body weight on Plenadren was −4.0 kg translating to a significant reduction in BMI, and waist circumference. A significant reduction was seen in HbA1c, but not fasting glucose, insulin or HOMA-IR. Hypoadrenal patients at baseline had significantly higher classical monocytes, lower non-classical monocytes and mature natural killer cells than the healthy controls. Whilst the patients on standard regimens showed no change, those who had switched to Plenadren showed normalisation of the affected immune cell populations, with their classical monocyte numbers coming down and the mature natural killer cell population rising. This coincided with a significantly better total infection scores and less flu-like illnesses in the patients on Plendaren, than those on standard regimens.

A DREAM ancillary study looked at PBMC clock gene expression in 26 of the Plenadren group, 29 of the standard treatment group and 16 of the healthy controls (49). At baseline, the hypoadrenal patients demonstrated altered expression of 19 genes including suppressed CLOCK, BMAL1 and elevated PER3 compared to the healthy controls. After 12 weeks of Plenadren, 18 of 19 genes were normalising to the levels of expression seen in the healthy volunteers. This indicates that the alterations in cellular timekeeping that are associated with traditional glucocorticoid replacement can be reversed with a more physiological glucocorticoid replacement profile.

Plenadren leads to an approximate 20% reduction in cortisol exposure in comparison to dose-matched standard hydrocortisone (38). It is distinctly possible that any notional benefits of Plenadren are solely due to this simple reduction in cortisol exposure and not necessarily due to the smoother pharmacokinetics (50). This exposure reduction in turn exposes patients to the risks of inadequate replacement, one manifestation of which may be higher fatigue scores recorded in subjective health scores (43). In order to mitigate against this, the current summary of product characteristics (SmPC) for Plenadren encourages individualisation of replacement doses when patients switch from other treatments (51). As a result, there is a trend towards escalation of the total daily dose in patients who have switched to Plenadren from real-world evidence (44, 45). By restoring the cortisol exposure to baseline with these dose increases, it is possible that the benefits to the surrogate endpoints seen in the randomised trials (which relied on 1:1 daily dose switching to Plenadren) may not be realised in routine clinical use.

Due to its formulation, Plenadren appears to be more vulnerable to malabsorption during intercurrent gastrointestinal disease. The sustained release preparation requires continued absorption of hydrocortisone from the gut for several hours, leading to diarrhoea as a common side effect. In addition to the caution for using Plenadren in chronic diarrhoea as noted above, there is a specific risk of hospitalisation with acute gastroenteritis (41) warranting particular mention in the SmPC as a situation where parenteral hydrocortisone may be needed (51).

## Prednisolone

Prednisolone, and its prodrug prednisone were first synthesised as anti-arthritic agents in 1950 (52). They have a similar chemical structure to cortisol with an additional double bond between carbon-1 and carbon-2 (C1–C2), which increases the half-life. Oral prednisone is converted to prednisolone during first-pass hepatic metabolism by 11β-hydroxysteroid dehydrogenase type 1 and for the purposes of this review, will be considered interchangeable with prednisolone (32). The pharmacokinetic and pharmacodynamic profile of prednisolone is notably different to cortisol. It has a 2.5 and 300 times greater binding affinity to both cortisol binding globulin and albumin, respectively, when compared to cortisol (53). *In vitro* studies have demonstrated that prednisolone has a greater affinity for the GR and mineralocorticoid receptor (MR) (54). When compared to cortisol, prednisolone binds 2.26 times more avidly at the level of the GR and 1.8 times at the MR. The increased affinity, may provoke downstream genomic effects due to the increased time needed for prednisolone to dissociate from the GR compared to hydrocortisone, which in turn slows the turning off of downstream transcription on a cellular level (55). It is difficult to quantify the cumulative effects of these individual differences between prednisolone and other glucocorticoids, underlining the need for clinical studies examining the global effects on clinical outcomes such as mortality.

The C1–C2 double bond endows prednisolone with a longer half-life of up to 3.2 h, and an increased potency when compared to cortisol (32). It has long been thought that the potency of prednisolone is four times greater than hydrocortisone and this may hold true at anti-inflammatory doses (56). However, there is emerging evidence that this value understates the actual bioequivalence at lower replacement doses. A cohort of 23 individuals with a median age of 9.4 years were treated with prednisolone for congenital adrenal hyperplasia, and compared to a cohort of 21 individuals with a median age of 8.3 years who were treated with thrice-daily hydrocortisone (57). Initially the prednisolone group were prescribed 2.4–3.75 mg/m^2^ of prednisolone once-daily, whilst the hydrocortisone group received 10–15 mg/m^2^ in keeping with the purported bioequivalence ratio of 4:1. In order to normalise the participants’ biochemical, clinical and anthropometric markers over the course of this 1-year study, the researchers were forced to reduce the amount of prednisolone prescribed on the study to 1.8–3.0 mg/m^2^, whilst the patients on hydrocortisone required an increase in dose to 12–20 mg/m^2^. The data from this study indicates that the potency of prednisolone may be as high as six to eight times greater than hydrocortisone, so that 3 mg of prednisolone is equivalent of 20 mg of hydrocortisone.

These findings raise questions about the true significance of studies comparing steroid replacement regimens where the ratio of 4:1 was used. In one study comparing prednisone 7.5 mg daily with hydrocortisone 30 mg daily, no difference was found between both groups in bone density (58). In a randomised, double-blind, placebo-controlled crossover study in Tunisia comparing prednisolone 5 mg and placebo versus twice-daily hydrocortisone 10 mg in fasting patients during Ramadan, there was no difference in glycaemic parameters and quality of life outcomes (59). A further study comparing 7.5 mg of prednisone with 27.8 mg of hydrocortisone found that patients on prednisone were predisposed to osteoporosis (60). It is, however, more likely that the adverse effects seen in this study were because the amount of prednisolone used was in fact equivalent to at least 45 mg of hydrocortisone, with bone turnover suppression having already been well characterised in increasing doses of hydrocortisone between 15 , 20 and 30 mg (61).

Arriving at the minimum required dose to maintain a patient with hypoadrenalism is difficult in the absence of established biomarkers. Slowly reducing the steroid dose can be dangerous but was inadvertently undertaken in a patient with secondary hypoadrenalism, where a replacement dose of 3 mg was found to be optimal (62). There has been progress in managing hypoadrenal patients with prednisolone following the development of mass spectrometry methods to quantify prednisolone levels (37). Whilst high pressure liquid chromatography methods have been available since the 1970s, they were limited by suboptimal recovery, interference and lower limits of quantification as high as 25 µg/L (63). Mass spectrometry has allowed for an improvement in detecting prednisolone with sensitivity as low as 10 µg/L, permitting higher resolution prednisolone day curves (37). Data from six such curves in hypoadrenal patients have shown that the prednisolone profile is remarkably similar to the cortisol profile produced by Plenadren (as shown in Fig. 2). Furthermore, the data from this study demonstrates that patients can be managed on lower doses of prednisolone than previously thought, with the 3.86 mg as the average dose used, and two patients using 3 mg. It also lays the groundwork for greater individualisation of dosing regimens for patients using prednisolone, guided by 8-h serum prednisolone levels.

Data comparing 82 patients on hydrocortisone and 64 on very low-dose prednisolone has shown no difference in most anthropometric and biochemical markers of metabolic risk, such as weight, blood pressure, lipid profiles including LDL and HDL, fasting glucose or HbA1c (65). Waist-hip ratio was lower and arbitrary satisfaction with prednisolone was higher, although this may be because of the convenience of once daily administration, as opposed to thrice-daily with hydrocortisone. This study was an early demonstration that very low dose prednisolone (2–4 mg) once daily may be useful in hypoadrenalism.

Prednisolone is particularly useful in adrenal insufficiency secondary to long-term steroid use. The same principles apply in inflammatory conditions where a slow wean of prednisolone is required in order to avoid resurgence of the initial condition (66). In these circumstances, the once-daily regimen of prednisolone allows for the formulation of easy weaning protocols that are simple for patients to adhere to, and to reverse where necessary. The use of prednisolone 1 mg tablets facilitates gradual reduction in dose. Such approaches are not possible with Plenadren as the lowest denomination available is 5 mg and the dual-release formulation prohibits splitting of tablets. The same approach with standard hydrocortisone, although possible, is hindered by the complexity of modifying thrice-daily regimens and the practical difficulty and imprecision from splitting the smallest available denomination of 10 mg tablets into smaller doses.

Although both prednisolone and hydrocortisone feature on the World Health Organisation list of essential medicines, prednisolone is more widely available. Higher doses of prednisolone are used for a number of anti-inflammatory indications. As a result, patients with hypoadrenalism are routinely managed with prednisolone in many countries, but at default doses of 5 mg that are higher than the 2–4 mg which limited evidence suggests constitutes adequate replacement. Dissemination of the message that three-quarters of a 5 mg tablet is sufficient for treatment of hypoadrenalism may well have an important impact on the health of patients across the world with hypoadrenalism.

## Conclusion

Emerging evidence strongly suggests that thrice-daily standard oral hydrocortisone has long-term deleterious effects. Our approach must centre on both preventing over-replacement and ensuring that there is appropriate steroid exposure at the correct times.

It may be that both Plenadren and prednisolone offer more suitable glucocorticoid replacement with concurrent cardiovascular, metabolic and immunological benefits, but there is a paucity of evidence directly comparing the two. There is also a lack of research comparing both prednisolone and Plenadren with other modalities of glucocorticoid replacement. Current studies are confounded by the relative differences in potency and dosing. Practically, both drugs offer a once-daily replacement with no current evidence of difference between the two. Further direct comparisons are therefore needed. It is hoped that ongoing trials such as PRED-AID and HYPER-AID will provide this (67, 68).

## Declaration of interest

The authors declare that there is no conflict of interest that could be perceived as prejudicing the impartiality of this review.

## Funding

SC is funded by a National Institute for Health Research http://dx.doi.org/10.13039/100005622 (NIHR), Doctoral Research Fellowship. TT is funded by the NIHR, NIHR BRC and the Moulton Charitable Research Foundation. KM is funded by the NIHR BRC. The views expressed are those of the authors and not necessarily those of the NHS, the NIHR or the Department of Health and Social Care.

## Author contribution statement

SC is responsible for the initial draft of this manuscript. TT, KL and KM have reviewed this manuscript and made edits to the text. All authors have approved the final manuscript. All authors have contributed equally.
